# The clinical assessment study of the foot (CASF): study protocol for a prospective observational study of foot pain and foot osteoarthritis in the general population

**DOI:** 10.1186/1757-1146-4-22

**Published:** 2011-09-05

**Authors:** Edward Roddy, Helen Myers, Martin J Thomas, Michelle Marshall, Deborah D'Cruz, Hylton B Menz, John Belcher, Sara Muller, George Peat

**Affiliations:** 1 Arthritis Research UK Primary Care Centre, Primary Care Sciences, Keele University, Staffordshire, ST5 5BG, UK; 2 Musculoskeletal Research Centre, Faculty of Health Sciences, La Trobe University, Bundoora, Victoria 3086, Australia

## Abstract

**Background:**

Symptomatic osteoarthritis (OA) affects approximately 10% of adults aged over 60 years. The foot joint complex is commonly affected by OA, yet there is relatively little research into OA of the foot, compared with other frequently affected sites such as the knee and hand. Existing epidemiological studies of foot OA have focussed predominantly on the first metatarsophalangeal joint at the expense of other joints. This three-year prospective population-based observational cohort study will describe the prevalence of symptomatic radiographic foot OA, relate its occurrence to symptoms, examination findings and life-style-factors, describe the natural history of foot OA, and examine how it presents to, and is diagnosed and managed in primary care.

**Methods:**

All adults aged 50 years and over registered with four general practices in North Staffordshire, UK, will be invited to participate in a postal Health Survey questionnaire. Respondents to the questionnaire who indicate that they have experienced foot pain in the preceding twelve months will be invited to attend a research clinic for a detailed clinical assessment. This assessment will consist of: clinical interview; physical examination; digital photography of both feet and ankles; plain x-rays of both feet, ankles and hands; ultrasound examination of the plantar fascia; anthropometric measurement; and a further self-complete questionnaire. Follow-up will be undertaken in consenting participants by postal questionnaire at 18 months (clinic attenders only) and three years (clinic attenders and survey participants), and also by review of medical records.

**Discussion:**

This three-year prospective epidemiological study will combine survey data, comprehensive clinical, x-ray and ultrasound assessment, and review of primary care records to identify radiographic phenotypes of foot OA in a population of community-dwelling older adults, and describe their impact on symptoms, function and clinical examination findings, and their presentation, diagnosis and management in primary care.

## Background

Symptomatic osteoarthritis (OA) is common in the general population, affecting the daily lives of an estimated 10% of people aged over 60 years [1]. It has a major impact on the quality of later life (OA is one of the ten leading causes of disability-adjusted life years [2]), on health care systems and costs (e.g. annual GP consultation rate of 250 per 10,000 persons aged 15 years and over [3]), and on economic productivity [4]. An ageing population and the rising prevalence of important causes of OA (e.g. obesity) ensure that it is an increasing challenge for the future [5].

The foot is the least studied joint complex affected by OA [6]. The prevalence of foot pain, problems and deformities (hallux valgus, arch deformities, hind-foot valgus) is high in community-dwelling older adults [7-12] and these contribute to locomotor disability [13-16], poor balance and risk of falling [17-19]. However, the contribution of foot OA within this is unclear. The first metatarsophalangeal joint (1^st ^MTPJ) was included in early descriptions of primary generalised OA [20], where it was shown to be relatively strongly associated with symptoms [21]. However, there are few examples internationally of epidemiological research that will extend our understanding of foot OA [6,22,23]. The recent publication of a validated atlas for scoring OA not only at the 1^st ^MTPJ but also at the 1^st ^and 2^nd ^cuneo-metatarsal joints (CMJ), the navicular-1^st ^cuneiform joint (NCJ) and the talo-navicular joint (TNJ) [24] now provides a basis for investigating patterns of radiographic foot OA, and their relation to impairment (e.g. pain and deformity), activity limitation and participation restriction.

The majority of ongoing formal healthcare for people with OA is provided in primary care. Peripheral joint pain is a common presentation to the primary care physician by older adults [25] and OA is one of the most frequently made diagnoses [26], yet there have been few systematic attempts to link defined clinical phenotypes with the diagnosis of OA in primary care [27]. Such research is needed to understand which phenotypes are seen by general practitioners, which are recognised as OA, and at what stage of development they are presented and recognised. Such research could form the basis for improved recognition, assessment and management of OA in primary care.

In addition to the questions of what phenotypes present to primary care and how they are managed, a crucially important issue is what effect primary care management has on outcome. Non-consultation for peripheral joint problems is common. Approximately 80% of those with musculoskeletal foot problems do not appear to consult their GP over prolonged periods of time (three years) [28]. Part of this is likely be related to the belief, pervasive among both the public and practitioners, that "nothing can be done". Furthermore, despite randomised controlled trial evidence about the short-term efficacy of primary care treatment for some peripheral joint problems [29], there are few investigations of the long-term effect of primary care consultation or OA management on impairment, activities or participation.

In summary, there is a paucity of research evidence concerning the radiographic phenotypes of foot OA and their impact on symptoms, clinical features, activity limitation and participation restriction. Important questions concerning how clinical phenotypes relate to the diagnosis of OA in primary care, and how the outcome from foot pain and OA is influenced by primary care consultation have been under-researched in relation to the foot but also at other joint sites. This prospective, observational, cohort study will combine unselected population sampling of older adults, self-reported survey data, comprehensive clinical and radiographic assessment, and linkage to computerised primary care records, to address these issues over a three-year period. It is designed to complement earlier studies of knee pain and OA [30] and hand pain, problems and OA [31] and permit combining of data across all three cohorts as well as direct comparison between them.

The aims of the study are to:

(i) Describe the frequency and pattern of co-occurrence of radiographic features of symptomatic OA in the following foot joints: the 1^st ^MTPJ, the 1^st ^and 2^nd ^CMJs, the NCJ and the TNJ.

(ii) Relate the occurrence of radiographic OA, described above, cross-sectionally to foot pain and disability, foot deformities, and soft tissue problems on physical examination. The associations between foot OA, foot pain, disability and footwear will also be examined.

(iii) Determine prospectively the factors that predict clinical deterioration, for example, radiographic OA, footwear characteristics, pain/OA at other sites, and psychosocial factors.

(iv) Identify which foot pain phenotypes present to primary care and are diagnosed in this setting.

(v) Describe the patterns of self-care and primary health care use for foot OA.

(vi) Model the effects of care on the outcome of severe foot pain.

## Methods

### Study design

**Figure 1:**
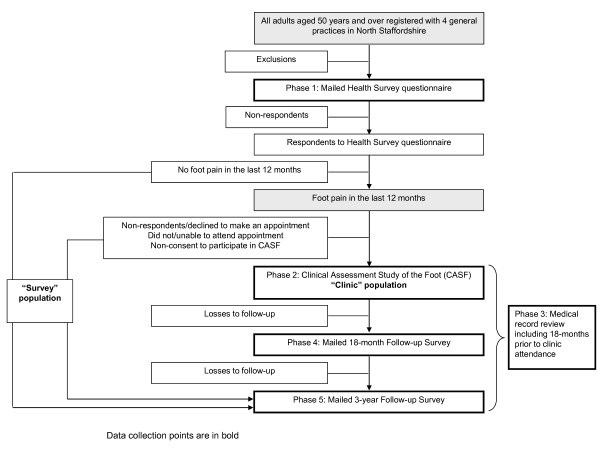
**Flowchart of study procedures**.

The study is a three-year population-based prospective observational cohort study. Ethical approval for all phases of the study has been obtained from Coventry Research Ethics Committee (REC reference number: 10/H1210/5). Adults aged 50 years and over registered with four separate local general practices will be invited to participate in the study, irrespective of consultation (Figure 1). Data collection will be in five phases:

Phase 1: Baseline postal Health Survey questionnaire

Phase 2: Baseline Clinical Assessment Study of the Foot (CASF)

Phase 3: Review of general practice medical records

Phase 4: Follow-up mailed survey at 18 months (Phase 2 participants only)

Phase 5: Follow-up mailed survey at 3 years (Phase 1 and Phase 2 participants)

### Phase 1: Baseline postal Health Survey questionnaire

All adults aged 50 years and over registered with four local general practices (mailed population approximately 9000 adults) will be mailed a letter of invitation from their general practitioner, a Participant Information Sheet, a Health Survey questionnaire, and a pre-paid return envelope. The lead general practitioner (GP) at each practice will be invited to identify potentially vulnerable patients (e.g. dementia, severe or terminal illnesses) they feel should be excluded from the study. Practice lists will be screened prior to mailing to ensure that addresses are up to date and exclude any recent deaths or departures from the practice list. Health Survey questionnaires will be mailed in batches (n = 500) to ensure regular recruitment to research clinics (Phase 2) and to limit the interval between questionnaire completion and clinic attendance. Pilot cognitive interviews have been undertaken with members of the Research Centre's Research User Group to test the Health Survey questionnaire's layout, readability, content, language and length. The questionnaire will be divided into five main sections: (i) general health (including generic measures of physical function, psychosocial factors and lifestyle [32-36] (Additional File 1: Appendix 1)); (ii) specific health problems including musculoskeletal co-morbidity and pain [37,38]; (iii) questions concerning the presence [39], duration, location [14], severity [40], and impact [41,42] of foot pain; (iv) demographic and socioeconomic characteristics [43,44]; and (v) employment (Table 1). Non-responders to the questionnaire will be sent a reminder postcard after two weeks. Those who do not respond to the reminder postcard will be sent a repeat questionnaire and Participant Information Sheet with a further covering letter four weeks after the initial mailing. Questionnaires will ask for consent (i) to contact participants again by post and/or (ii) to review medical records. Responders will be given the option of providing their telephone number for further contact.

**Table 1:** Content of baseline postal Health Survey questionnaire

Concept	Measurement method	Detail
**Section A: General health**		
Perceived general health	MOS SF12 [33]	Physical and mental component summary scores
Physical function	MOS SF36 [32]	Physical functioning sub-scale
Anxiety and depression	Hospital anxiety and depression scale [34]	Anxiety and depression sub-scales
Participation	Keele Assessment of Participation (KAP) [35,73]	5-items assessing person-perceived, performance-based participation
Support	Emotional support: single question	Yes, no, no need
	Physical support: single question	Yes, no, no need
Life-style	Smoking status	Current, previous, never
Anthropometric characteristics	Self-reported height	
	Self-reported weight	
Footwear	Toe-box breadth line drawings (Additional File 1: Appendix 1)	Type most frequently worn by decade
	Heel height line drawings (females only) (Additional File 1: Appendix 1)	Type most frequently worn by decade
Physical activity	Short-Form International Physical Activity Questionnaire (IPAQ) [36]	Frequency and duration of 4 activities performed during previous 7 days
**Section B: Specific health problems**		
Hallux valgus	Self-completed line drawings [37]	5 line-drawings for each foot depicting increasing severity of hallux valgus
Co-morbidities	Falls, fractures, chest problems, heart problems, deafness, problem with eyesight, raised blood pressure, diabetes, stroke, cancer, liver disease, kidney disease, poor circulation, rheumatoid arthritis	Yes, for any that apply
Intermittent claudication	Edinburgh Claudication Questionnaire [38]	Pain or discomfort in legs when walking, pain characteristics, pain location (leg manikin)
Bodily pain	Self-completed body manikin	In the past 4 weeks, have you had pain that has lasted for one day or longer in any part of your body? If yes, shade pain location on manikin
	Site-specific questions	Have you had any problems with your hands or pain in your hands/hips/knees/feet in the last year?
**Section C: Foot pain**		
Foot pain characteristics	Side of pain	Both, right, left
	Duration in past 12 months	< 7 days, 1-4 weeks, 1-3 months, 3+ months
	Foot injury: Have you ever injured your foot badly enough to see a doctor about it?	No, right foot only, left foot only, both feet
	Foot pain, aching, stiffness in last month [39]	No days, few days, some days, most days, all days
	Location: self-completed foot manikin [14]	In the past month, have you had any ache or pain that has lasted for one day or longer in your feet? If yes, shade pain location on foot manikin
	Foot pain intensity in last month [40]	0-10 NRS with verbal anchors (no pain, pain as bad as can be)
Complaint-specific functioning	Manchester Foot Pain and Disability Index [41]	19-items across four constructs: pain, function, appearance, work/leisure
Coping strategies for foot pain	Foot-related fatigue: single item Single-item coping strategies questionnaire [42]	None of the time, on some days, on most/every day(s) 0-6 NRS with verbal anchors (never do that, always do that)
Healthcare use	Medication use in last month	For foot pain, for other pain
	Consultation in last 12 months for foot pain	General practitioner, physiotherapist, podiatrist, chiropodist (NHS and private)
**Section D: Demographic/socioeconomic characteristics**		
Demographic characteristics	Date of birth Gender	
	Marital status	Married, separated, divorced, widowed, cohabiting, single
	Living arrangements	Alone, not alone
Socioeconomic characteristics	Current employment status	Employed, not working due to ill-health, retired, unemployed/seeking work, housewife, other
	Current/recent job title	Free text
	Current/recent job title of spouse	Free text
	Adequacy of income [43]	Find it a strain to get by from week to week, have to be careful with money, able to manage without much difficulty, quite comfortably off
	Higher education	Yes/no (If yes, age finished full-time education)
	Ethnicity	White UK/European, Afro Caribbean, Chinese, Asian, African, Other
**Section E: Work**		
	Work status	Working full-time, part time, or off work due to ill-health
	Work performance	0-10 NRS with verbal anchors (worst performance, best performance)
	Work limitation due to a health problem or physical limitation	Not affected, changed the way I do the job, reduced the number of hours, currently off work
	Job lock	Would like to leave work but can't due to financial needs

MOS SF 12 = Medical Outcomes Study Short Form 12; MOS SF 36 = Medical Outcomes Study Short Form 36; NRS = numerical rating scale

### Phase 2: Baseline Clinical Assessment Study of the Foot (CASF)

Responders to the Health Survey questionnaire who report experiencing pain in or around the foot within the last twelve months and who provide written consent to further contact will be sent a letter of invitation to attend a research clinic. The letter of invitation will be accompanied by a Participant Information Sheet providing details of the study. Participants will be asked to telephone the Research Centre if they are interested in taking part in order to book an appointment. Non-responders to this initial invitation letter will be sent a reminder invitation approximately two weeks later.

Those willing to take part in the study will be booked into the next convenient appointment and, if necessary, travel arrangements (taxi) made. Postal confirmation of the appointment will be made by letter and then by a reminder postcard shortly prior to the appointment. The postcard will be mailed in an envelope to maintain confidentiality about the nature of the appointment. Participants who do not attend the clinic for their specified appointment will be sent another letter asking them to re-contact the Research Centre and book another appointment if they still wish to participate.

Assessment clinics for the study will be conducted twice-weekly in a local NHS Trust community rheumatology hospital. A maximum of 12 appointments per week are scheduled. Each clinic is to be staffed by a Clinic Co-ordinator, a Clinic Support Worker, two trained Health Professionals (podiatrist or physiotherapist) acting as Research Assessors, one trained Research Assessor (physiotherapist, radiographer or nurse) acting as an Ultrasonographer, and two Radiographers.

On arriving at clinic, participants will be issued with a file containing all assessment documentation marked with their unique study number. Prior to commencing the assessment, the procedures outlined in the Participant Information Sheet will be discussed with each participant. Participants will be given the opportunity to ask questions. Written informed consent to take part in the study will be obtained from all participants. Appropriate clothing (shorts) for the assessment will be provided.

Participants will undertake the following standardised assessment: digital photography of both feet and ankles; plain radiography of both feet, ankles and hands; ultrasound of the plantar fascia in both feet; clinical interview; physical assessment of the feet, lower limb and hands; simple anthropometric measurement and self-complete questionnaire (Table 2). Each participant's visit is expected to last approximately 2 hours.

**Table 2:** Content of clinical assessment: clinical interview, physical examination and self-complete questionnaire

Concept	Measurement method	Detail
**Clinical Interview**		
Pre-assessment screening:		
Screen for clinical "red flags"	Recent significant foot or hand injury Recent sudden change in foot symptoms	
Screen for joint surgery	History of joint operations	
Foot pain characteristics	Side of pain	
	Comparative severity of bilateral symptoms	
	Duration	Within 12 months, 1-5 years, 5-10 years, 10+ years (for each foot)
	Preceding accident/injury	Yes/no
	Foot pain/aching/discomfort in last month	Yes/no
Foot pain quality	Short-form McGill Pain Questionnaire [47]	15 sensory and affective descriptors
Sleep disturbance	Self-report	Yes/no
Sensory disturbance	Self-reported tingling/numbness/pins and needles	Yes/no (for each foot)
Causal attribution	What do you think has caused the problem with your foot/feet?	Recorded verbatim
Diagnostic attribution	What do you think is the matter with your foot/feet now?	Recorded verbatim
Foot surgery	Details of any foot surgery	Nature of surgery Right/left < 1 year, 1- < 5 years, 5- < 10 years, 10+ years ago
Foot/ankle injury	Details of foot/ankle injury	Sprain, fracture, other Right/left; forefoot, mid-foot, heel, ankle < 1 year, 1- < 5 years, 5- < 10 years, 10+ years ago
Planned treatment	Are you waiting for any appointments or treatments for this foot or ankle problem?	Yes/no (free text comments for yes)
Importance of health problems	What would you consider to be your two most important health problems at the moment? *[Includes foot problem]*	Recorded verbatim
		
**Physical examination**		
Screen for clinical "red flags"	Acutely, swollen, hot, painful feet or hands	Yes/no (free text for comments)
Observation	Skin lesions	Bunionette, hyperkeratotic lesions, ulcers (plantar and dorsal aspect)
Toe deformity	MTPJ and interphalangeal joint hyperextension Mallet toe, hammer toe, claw toe, retracted toe	Present/absent (great toe) Present/absent (lesser toes)
Palpation	Mid-foot bony exostosis Plantar fascia tenderness	Present/absent Present/absent (insertion and mid-arch)
Foot posture	Foot Posture Index [50]	Six-criterion scoring system
	Navicular Height [49]	Millimetres
	Foot Length [51]	Millimetres
	Arch index [48,49]	Weightbearing footprint. Length of footprint excluding toes is divided into equal thirds. Arch index = area of middle third divided by area of entire footprint
Range of movement (foot/ankle)	Ankle dorsiflexion (with knee flexed and extended) [53]	Degrees
	Subtalar inversion [52]	Degrees
	Subtalar eversion [52]	Degrees
	1^st ^MTP joint dorsiflexion [54]	Degrees
Knee valgus/varus deformity	Intercondylar distance	Centimetres
	Intermalleolar distance	Centimetres
Anthropometric measurements	Height	Metres
	Weight	Kilograms
Lower limb physical function	Short physical performance battery (SPPB) [57]	Standing balance test, timed repeated chair stand test, 4-metre gait speed test
Hand osteoarthritis	Deformity, enlargement, swelling, nodes [55]	Observation and palpation: swelling (MCPJ), nodes (PIPJ and DIPJ), deformity and enlargement (1^st ^CMCJ, PIPJ and DIPJ)
Hand function	Power grip strength (Jamar dynanometer) [56]	Pounds
	Pinch grip strength (B&L pinch gauge) [56]	Pounds
		
**Self-complete questionnaire**		
***Section A: Foot Pain***		
Foot pain chronicity	Chronic Pain Grade [59]	6 questions (0-10 NRS) and 1 question (4 response options) giving grade I-IV
Complaint-specific functioning	Symptom satisfaction [64]	5-point Likert scale (Very dissatisfied to Very satisfied)
***Section B: Hand pain and problems***		
Hand pain characteristics	Hand pain in last 12 months Side of pain Duration in past 12 months Hand pain, aching, stiffness in last month [55]	Present/absent < 7 days, 1-4 weeks, 1-3 months, 3+ months No days, few days, some days, most days, all days

	Hand pain intensity in last month [40]	0-10 NRS with verbal anchors (no pain, pain as bad as could be)
	Location: self-completed hand manikin [60] AUSCAN [65,66]	In the past month, have you had any ache or pain that has lasted for one day or longer in your hand? If yes, shade location on hand manikin
		Pain and stiffness sub-scales
Complaint-specific functioning	AUSCAN [65,66]	Physical function sub-scale
Hand dominance	Self-report	Right, left, both
Healthcare use	GP consultation within last 12 months for hand problem	
***Section C: Hip pain***		
Hip pain characteristics	Side of pain	Both, right, left
	Duration in past 12 months	< 7 days, 1-4 weeks, 1-3 months, 3+ months
	Hip pain, aching, stiffness in last month [61]	No days, few days, some days, most days, all days.
	WOMAC (hip) [62]	Pain and stiffness sub-scales
Complaint-specific functioning	WOMAC (hip) [62]	Physical function sub-scale
Healthcare use	GP consultation within last 12 months for hip pain	
***Section D: Knee pain***		
Knee pain characteristics	Side of pain	Both, right, left
	Duration in past 12 months	< 7 days, 1-4 weeks, 1-3 months, 3+ months
	Knee pain, aching, stiffness in last month [63]	No days, few days, some days, most days, all days.
	WOMAC (knee) [62]	Pain and stiffness sub-scales
Complaint-specific functioning	WOMAC (knee) [62]	Physical function sub-scale
Healthcare use	GP consultation within last 12 months for knee pain	

AUSCAN = Australian Canadian Osteoarthritis Hand Index; CMCJ = carpometacarpal joint; DIPJ = distal interphalangeal joint; GP = General practice; MTPJ **= **metatarsophalangeal joint; MCPJ = metacarpophalangeal joint; NRS = numeric rating scale; PIPJ = proximal interphalangeal joint; WOMAC = Western Ontario and McMaster Universities Osteoarthritis Index

#### Digital photography

Each participant will have three photographs taken by a Research Assessor using a digital camera (Canon Digital IXUS 75: Resolution 7.1 mega pixels, 3× zoom). Each foot will be imaged separately with the participant standing in a specially designed mirror-box that enables images of the dorsum, medial and lateral aspects of their foot to be captured in a single photograph. An additional posterior view photograph of both feet will be taken with the participant positioned in a self-selected relaxed bipedal stance on a gym step using a separate camera (Canon PowerShot A480: Resolution 10.0 mega pixels 3.3× zoom) mounted on a tripod to the height of the step. The photograph will be taken at a distance of 40 cm and will capture the heels, ankles and lower limb. To preserve anonymity participants' faces will not be included in any of the photographs: their unique study number will be placed in each frame. Permission to use anonymised digital images for educational purposes will be sought in the written consent form. Digital photography will take approximately 5 minutes to complete for each participant.

#### Plain radiography of the feet and hands

Digital radiographs of both feet, ankles and hands will be obtained for all participants. Weight-bearing dorso-plantar and lateral views of each foot will be obtained according to a defined protocol [24] and stored on disc. The participant will stand in a relaxed position with the weight of the participant's body distributed equally. A relaxed position will be achieved by asking the participant to walk on the spot for a few steps and then stand relaxed. For the dorso-plantar view the participant will stand with the plantar aspect of both feet on the detector. The x-ray tube will be angled 15° cranially with a vertical central ray centred at the base of the third metatarsal [24]. For lateral projections the participant will stand on a low platform with the detector positioned at the side of the participant's foot. The x-ray tube will be angled at 90° with a horizontal central ray centred on the base on the base of the first metatarsal [24]. Weight-bearing antero-posterior views of both ankle joints will also be obtained with the participant standing on the low platform. The detector will be positioned behind the participant. The x-ray tube will be angled 90° with a horizontal central ray centred midway between the malleoli [45]. Dorso-palmar views of both hands are to be performed. The palmar aspect of the hand will be placed on the detector with the fingers extended, separated slightly and spaced evenly [31]. A vertical central ray will be centred on the head of the third metacarpal [45]. Each foot, ankle and hand will be imaged separately and the film focus distance will be set at 110 cm for all projections. X-rays will take approximately 20 minutes to complete for each participant.

#### Ultrasound of the plantar fascia

The ultrasound examination will be performed using a variable frequency 8-13 MHz linear transducer with a Logiq-e ultrasound system (GE Healthcare). The participant will be positioned in a self-selected half-lying position, or sitting position if the half-lying position cannot be assumed by the participant, on a couch with their feet hanging over the end of the couch and ankles dorsiflexed to 90 degrees. Real-time sagittal (longitudinal) imaging of the plantar aponeurosis will be performed with the focus adjusted to the depth of the fascia for each participant. Plantar fascia thickness will be measured at a standard reference point where the plantar fascia crosses the anterior aspect of the inferior border of the calcaneus on the longitudinal view but at its thickest point in the transverse plane [46]. Three measurements will be taken and recorded on a paper proforma. The Research Assessor performing the ultrasound will be blind to the results of the clinical assessment. The scan will take approximately 10 minutes for each participant.

Ultrasound images will be retained and digitally stored at the Research Centre for quality control purposes. Consent will be sought in the clinic consent process for the use of anonymised images for educational purposes and in presentations.

#### Clinical interview and physical examination

Participants will be interviewed and examined by a trained Research Assessor who will be blind to the radiographic and sonographic findings. This procedure will comprise three components. Firstly, a standardised clinical interview will be conducted to gather quantitative data relating to foot pain and symptoms in older adults [47], causal and diagnostic attribution, previous injury or surgery, and planned treatment (Table 2). Secondly, a detailed, standardised, examination of both feet will be conducted. This will include assessment of skin lesions; common deformities; foot posture including static arch index [48,49], Foot Posture Index [50], foot length [51], navicular height [49,51]; and range of movement of subtalar inversion and eversion [52], ankle dorsiflexion [53], and 1^st ^MTPJ dorsiflexion [54] (Table 2). Thirdly, a brief standardised physical examination of both hands, and both knees will be conducted (Table 2). This will include assessment of presence of deformity, enlargement, swelling and nodes in both hands [55]; maximal power and pinch grip strength using a Jamar dynamometer and B&L pinch gauge respectively [56]; and presence of varus and valgus deformities at the both knees. Lower extremity physical performance will be also assessed [57].

Plantar pressures from both feet will be recorded during level barefoot walking using a pressure platform (RS Scan^® ^International, Olen, Belgium). This system consists of a 12 mm thick floor mat (578 mm × 418 mm) incorporating 4096 resistive sensors sampling at a rate of 300 Hz. The two-step gait initiation protocol will be used whereby the participant is positioned two step lengths from the front edge of the pressure platform and is instructed to walk in a normal manner, striking the sensor area with the second step [58]. The system will be calibrated at the beginning of each session and recalibrated for participants' individual weight and shoe size prior to each assessment. The participant will complete several practice trials, to allow them to familiarise themselves with the two step approach and calculate their starting position. Three trials will be recorded for each foot. Maximum force (N), peak pressure (N/cm^2^) and contact time (ms) will be collected. Footprints obtained will be divided into masks corresponding to the major structural regions of the foot.

Pre-defined protocols for all components of the interview and assessment will be used for standardisation between Research Assessors. Assessment findings will be recorded on a standard form that is to be checked for missing data immediately post-assessment by the Clinic Co-ordinator or Clinic Support Worker. Discussion between Research Assessors and participants about diagnosis and/or appropriate management will be discouraged. Participants will be advised to discuss clinical queries with their General Practitioner. The interview and assessment will take approximately 40 minutes to complete for each participant.

#### Simple anthropometric measurements

Weight (in kg) and height (in cm) of each participant will be measured using calibrated digital scales (Seca Ltd., Birmingham, UK) and a wall-mounted measure (Seca Ltd., Birmingham, UK) respectively.

#### Self-complete clinic questionnaire

During the clinic visit, participants will complete a self-complete questionnaire. The questionnaire will be divided into four main sections: (A) Foot pain; (B) Hand pain and problems; (C) Hip pain; and (D) Knee pain. Questions will relate to pain [40,55,59-63], site-specific function [62,64-66], and GP consultation (Table 2). Section A will be completed by all clinic-attenders. Sections B, C, and D will be completed only by those who reported hand, hip or knee pain respectively in their Health Survey questionnaire. The Clinic Co-ordinator or Clinic Support Worker will guide participants as to which sections need to be completed and will check all questionnaires following completion for any missing data. The questionnaire will take approximately 30 minutes to complete.

Travelling and out-of-pocket expenses will be reimbursed after the assessment.

#### Post-clinic procedure

The digital cameras, study laptop and all completed clinical assessment documentation and questionnaires will be returned to the Research Centre. Digital images will be downloaded from the memory cards and laptop onto a secure server.

A clinical report on the x-ray images will be provided by a Consultant Radiologist at the NHS Trust Hospital. The images and report will be forwarded to the Research Centre where they will be screened by a Consultant Rheumatologist for any radiographic "red flags" or significant radiographic abnormality (see below).

Standardised coding of radiographic features on the foot and hand x-ray images will be carried out by the Research Radiographer (a trained observer with a background in diagnostic radiography). The Research Radiographer will be blinded to all assessment data and the radiologist's report. Foot images will be scored for individual radiographic features, including osteophytes and joint space width, at the 1^st ^MTPJ, 1^st ^and 2^nd ^CMJs, NCJ and TNJ according to the Menz atlas and classification system [24]. With the exception of the TNJ, both dorso-plantar and lateral projections will be used to assess osteophyte and joint space width. For the grading of TNJ osteophytes, only the lateral projection will be used as the dorsal aspect of the joint, where osteophytes most commonly develop, is not easily visualised on the dorso-plantar projection. Standardised coding of radiographic features using the Kellgren and Lawrence grading system will be completed for the ankle joints and sixteen joints in each hand and wrist [67]: the distal interphalangeal joints (DIP), the proximal interphalangeal joints (PIP), the interphalangeal joint of the thumb (IP), the metacarpophalangeal joints (MCP), the thumb carpometacarpal joint (CMC) and the trapezioscaphoid joint (TS).

Consent forms, assessment documentation, digital x-ray images and reports are to be placed in separate secure storage.

#### Communication with participants' general practice

Assessment findings will be communicated to participants and their General Practice only in specific circumstances that will be explained to participants at the start of the clinic:

#### Mandatory notification of clinical 'red flags'

All participants will be routinely screened during the clinical assessment for signs and symptoms suggesting potentially serious pathology requiring urgent medical attention (Table 2). These are: recent trauma to the feet or hands that may have resulted in significant tissue damage; recent sudden worsening of foot or hand symptoms; and acutely hot, swollen, painful feet or hands [68]. In the event of such findings, participants will be informed that they require urgent attention, a standard fax will be immediately sent to the General Practice, and appropriate medical attention arranged the same day. A letter of confirmation will be subsequently sent to the participants' General Practice.

#### Mandatory notification of radiographic 'red flags'

In the event of any radiographic red flags (including suspected malignancy, unresolved fracture, infection) reported by the Consultant Radiologist a standard fax will be sent with a copy of the x-ray report to the General Practice notifying them of this. This will subsequently be confirmed by letter.

#### Discretionary notification of other significant radiographic abnormality

At the discretion of the Consultant Rheumatologist, the General Practice will be notified of other significant radiographic abnormality (e.g. previous fracture, inflammatory arthropathy).

#### Availability of x-ray report on request

To prevent unnecessary duplication of x-rays, participants' GPs can request an x-ray report if they feel it would be valuable for clinical management.

#### Quality assurance and control

Quality assurance and control are important for the integrity of longitudinal studies and the validity of their conclusions [69]. This is especially true of observer-dependent methods of data-gathering. In the clinical assessment phase of the study, the clinical interview and physical assessment, ultrasound, digital images, plantar pressure and the taking and scoring of x-ray will be subject to a number of quality control procedures.

Inter- and intra-assessor reliability of foot interview and examination variables have been established, where possible, from the published literature [49-54,70]. Assessors will undergo training in consent procedures, clinical interview and physical assessment techniques. All Research Assessors will be required to conduct at least two clinical assessments prior to the commencement of data collection. During the first month clinics with reduced numbers of participants will be held to allow all study procedures to be tested and reviewed. All radiographers participating in the study will also receive training prior to the commencement of the study.

Selected Research Assessors will receive ultrasound training on a formally assessed course, Focused Specialist Ultrasound Practice, run by University of Derby (UK). This course consists of the principles of ultrasound physics and imaging science. The Research Assessors will then receive specific clinical training from a Consultant Musculoskeletal Sonographer to assess the plantar fascia thickness. In addition to meeting the course assessment requirements clinical competence for the study will be assessed by the Consultant Sonographer following a period of supervision and mentorship.

The Research Radiographer will be trained in the methods for scoring the plain radiographs. This single observer will score all images and intra-observer variability will be assessed using 60 sets of images scored eight weeks apart. Inter-observer variability will be assessed using a second observer with prior experience of grading foot x-rays for OA who will also grade 60 sets of images.

A detailed Assessor Manual with protocols for obtaining written informed consent, digital photography, clinical interview and physical assessment, administration of the self-complete questionnaire, anthropometric measurement, plain radiography, and ultrasound will be provided to all members of the study team for reference during the entire study period.

During the data collection period, digital photographs for all participants will be reviewed and participants with any missing or spoilt images will be recalled to repeat the photographs. Quality control sessions for consent procedures, clinical interview and physical assessment, radiography and ultrasound will be undertaken at regular intervals throughout the study. These sessions will include observation of assessments in clinic by the Principal Investigator, structured observation of assessments in a healthy volunteer, and direct inter-assessor comparisons on selected participants. Observation of radiography and ultrasound will be undertaken by the Research Radiographer and Consultant Musculoskeletal Sonographer respectively. The outcome of each quality control session will be fed back to the individual Research Assessor and the group as a whole.

### Phase 3: Review of general practice medical records

All participants in Phase 1 who give permission for their GP records to be accessed will have their computerised medical records tagged by a member of the Research Centre's Health Informatics Specialist team. All consultations for the 18-month period prior to clinic attendance, and for the three-year period following clinic attendance, will be identified. The four practices participating in this study are fully computerised and undergo annual audits completed by the Health Informatics team to assess the quality and completeness of the data entry at the practices [71].

This data will cover consultations, prescriptions, and referrals. All relevant foot-related consultations will be identified using search techniques based on Read codes and free text entries, which have been previously developed and successfully applied by the Research Centre [28,72]. Participants with a relevant recorded consultation will be classified into those receiving an OA diagnosis recorded by their GP and those receiving non-specific symptom codes (e.g. arthralgia). In addition, all comorbid consultations will be identified and sub-grouped by Read code chapter.

Patterns of primary and secondary health care utilisation will be compared between Phase 2 participants and Phase 1 participants who did not attend the research clinic. All sensitive data (name, contact details) will be removed from the medical records data and the consultation data will be linked to the survey and clinical assessment data by unique survey identifier.

### Phase 4: Follow-up mailed survey at 18 months (Phase 2 respondents only)

Follow-up surveys will be mailed to all Phase 2 participants 18 months after their baseline clinical assessment. The focus of follow-up will be clinical (severity of pain and functional limitation) change and possible determinants of this. The content of this survey is provided in Table 3. Non-responders to the questionnaire will be sent a reminder postcard after two weeks. Those who do not respond to the reminder postcard will be sent a repeat questionnaire and Participant Information Sheet with a further covering letter four weeks after the initial mailing. Primary outcome data will be sought from non-respondents by telephone interview or shortened postal questionnaire. We plan to trace participants who have moved practice during the follow-up period using the NHS tracing service.

**Table 3:** Content of 18-month postal follow-up Health Survey questionnaire (Phase 2 participants only)

Concept	Measurement method	Detail
Foot pain characteristics	Change in foot pain over past 18 months	Completely recovered, much better, better, no change, worse, much worse
	Since your assessment 18 months ago, have you ever injured your foot badly enough to see a doctor about it?	No/right only/left only/both
	Foot pain, aching, stiffness in last month [39]	No days, few days, some days, most days, all days
	Foot pain intensity in past month [40]	0-10 NRS with verbal anchors (no pain, pain as bad as could be)
Foot pain chronicity	Chronic Pain Grade [59]	6 questions (0-10 NRS) and 1 question (4 response options) giving grade I-IV
Complaint-specific functioning	Manchester Foot Pain and Disability Index [41]	19-items across four constructs: pain, function, appearance, work/leisure
	Symptom satisfaction [64]	5-point Likert scale (Very dissatisfied to Very satisfied)
Healthcare use	Use of services/treatments for foot pain in past 18 months	GP, physiotherapist, hospital specialist, acupuncture, podiatrist, chiropodist, drugs on prescription, foot injection, foot surgery, osteopath/chiropractor, other (specify)
	Medication use in last month	For foot pain, for other pain
Coping strategies for foot pain	Single-item coping strategies questionnaire [42]	0-6 NRS with verbal anchors (never do that, always do that)
Perceived general health	MOS SF 12 [33]	Physical and mental component summary scores
Physical function	MOS SF 36 [32]	Physical functioning sub-scale
Anxiety and depression	Hospital anxiety and depression scale [34]	Anxiety and depression sub-scales
Hallux valgus	Self-completed line drawings [37]	5 line-drawings for each foot depicting increasing severity of hallux valgus
Bodily pain	Self-completed body manikin	In the past 4 weeks, have you had pain that has lasted for one day or longer in any part of your body? If yes, shade location of pain on manikin
Regional pain	Site-specific questions	Have you had any problems with your hands or pain in your hands/hips/knees in the last year?
Demographic characteristics	Date of birth Gender	
Socioeconomic characteristics	Current employment status	Employed, not working due to ill-health, retired, unemployed/seeking work, housewife, other

MOS SF 12 = Medical Outcomes Study Short Form 12; MOS SF 36 = Medical Outcomes Study Short Form 36; NRS = numerical rating scale

### Phase 5: Follow-up mailed survey at 3 years (Phase 1 and Phase 2 respondents)

Follow-up surveys will be mailed to all Phase 1 and 2 participants 3 years after their baseline Health Survey questionnaire. In addition to information about clinical change in Phase 2 participants, the survey will also include repeat measures of lifestyle [36,73], general health (including generic measures of physical function [32,33]), psychosocial factors [34], co-morbidity [37,38] and basic screening questions concerning the presence [39], duration, location [14], severity [40], and impact of foot pain [41,42] (Table 4). Non-responders to the questionnaire will be sent a reminder postcard after two weeks. Those who do not respond to the reminder postcard will be sent a repeat questionnaire and Participant Information Sheet with a further covering letter four weeks after the initial mailing. Primary outcome data will be sought from non-respondents by telephone interview (Phase 2 participants only) or shortened postal questionnaire. We plan to trace participants who have moved practice during the follow-up period using the NHS tracing service.

**Table 4:** Content of 3-year postal follow-up Health Survey questionnaire (Phase 1 and Phase 2 participants)

Concept	Measurement method	Detail
**Section A: General health**		
Perceived general health	MOS SF 12 [33]	Physical and mental component summary scores
Physical function	MOS SF 36 [32]	Physical functioning sub-scale
Anxiety and depression	Hospital anxiety and depression scale [34]	Anxiety and depression sub-scales
Participation	Keele Assessment of Participation (KAP) [35,73]	5-items assessing person-perceived, performance-based participation
Physical activity	Short-Form International Physical Activity Questionnaire (IPAQ) [36]	Frequency and duration of 4 activities performed during previous 7 days
		
**Section B: Specific health problems**		
Hallux valgus	Self-completed line drawings [37]	5 line-drawings for each foot depicting increasing severity of hallux valgus
Co-morbidities	Falls, fractures, chest problems, heart problems, deafness, problems with eyesight, raised blood pressure, diabetes, stroke, cancer, liver disease, kidney disease, poor circulation, rheumatoid arthritis	Yes for any that apply
Intermittent claudication	Edinburgh Claudication Questionnaire [38]	Pain or discomfort in legs when walking, pain characteristics, pain location (leg manikin)
Bodily pain	Self-completed body manikin	In the past 4 weeks, have you had pain that has lasted for one day or longer in any part of your body? If yes, shade location of pain on manikin
	Site-specific questions	Have you had any problems with your hands or pain in your hands/hips/knees/feet in the last year?
		
**Section C: Foot pain**		
Foot pain characteristics	Side of pain	Both, right, left
	Duration in past 12 months	< 7 days, 1-4 weeks, 1-3 months, 3+ months
	Have you ever injured your foot badly enough to see a doctor about it?	No/right foot only/left foot only/both feet
	Location: self-completed foot manikin [14]	In the past month, have you had any ache or pain that has lasted for one day or longer in your feet? If yes, shade location of pain on foot manikin
	Foot pain, aching, stiffness in last month [39]	No days, few days, some days, most days, all days
	Foot pain intensity in last month [40]	0-10 NRS with verbal anchors (no pain, pain as bad as could be)
Complaint-specific functioning	Manchester Foot Pain and Disability Index [41]	19-items across four constructs: pain, function, appearance, work/leisure
Coping strategies for foot pain	Single-item coping strategies questionnaire [42]	0-6 NRS with verbal anchors (never do that, always do that)
Healthcare use	Medication use in last month	For foot pain, for other pain
	Consultation with general practitioner in last 12 months for foot pain	
		
**Section D: Demographic/socioeconomic characteristics**		
Demographic characteristics	Date of birth Gender	
	Marital status	Married, separated, divorced, widowed, cohabiting, single
	Living arrangements	Alone, not alone
Anthropometric characteristics	Self-reported height	
	Self-reported weight	
Socioeconomic characteristics	Current employment status	Employed, not working due to ill-health, retired, unemployed/seeking work, housewife, other
	Current/recent job title	Free text
	Current/recent job title of spouse	Free text
	Adequacy of income [43]	Find it a strain to get by from week to week, have to be careful with money, able to manage without much difficulty, quite comfortably off
**Additional questions for phase 2 participants only**		
Foot pain characteristics	Change in foot pain over past 3 years	Completely recovered, much better, better, no change, worse, much worse
	Since your assessment 3 years ago, have you injured your foot badly enough to see a doctor about it?	No/right foot only/left foot only/both feet
Foot pain severity	Chronic Pain Grade [59]	6 questions (0-10 NRS) and 1 question (4 response options) giving grade I-IV
	Symptom satisfaction [64]	5-point Likert scale (Very dissatisfied to Very satisfied)
Healthcare use	Use of services/treatments for foot pain in past 3 years	GP, physiotherapist, hospital specialist, acupuncture, podiatrist, chiropodist, drugs on prescription, foot injection, foot surgery, osteopath/chiropractor, other (specify)

MOS SF 12 = Medical Outcomes Study Short Form 12; MOS SF 36 = Medical Outcomes Study Short Form 36; NRS = numerical rating scale

### Sample size

The sample size for this study was determined by the estimated numbers of participants needed in Phase 2 in order to ensure sufficient power for both cross-sectional and longitudinal analyses. The primary aim is to compare the proportion of participants with poor functional outcome across the radiographic (p_2_) and no radiographic OA groups (p_1_) at 3 years. Assuming p_1 _= 20% in the unexposed group, a sample size of 426 will have 80% power to detect a relative risk (p_2_/p_1_) of 1.62 using a 5% significance level. Allowing for a drop-out figure of 80 from baseline to three years will require an initial recruitment of 506 participants to Phase 2.

### Statistical analysis

#### Patterns of symptomatic radiographic foot OA

The frequency and co-occurrence of radiographic features of symptomatic OA at the 1^st ^MTPJ, the 1^st ^and 2^nd ^CMJs, the NCJ and the TNJ will be described using simple descriptive statistics.

#### Features associated with foot OA phenotypes

Linking data collected at the clinical assessment with that from the baseline health survey questionnaire, the occurrence of radiographic OA will be related cross-sectionally to foot pain and disability, foot deformities, soft-tissue problems, footwear characteristics and pain/OA at other sites, using odds ratios and associated 95% confidence intervals adjusted for age, gender and BMI. The effect of missing primary outcome data will be investigated using multiple imputation methods.

#### Outcome of foot OA at 3 years

Linking baseline date to 18-month and three-year follow-up questionnaires, we will then be able to determine prospectively the factors that are related to clinical deterioration using risk ratios and associated 95% confidence intervals, for example, radiographic OA, footwear characteristics, pain/OA at other sites, psychosocial factors.

#### The presentation and diagnosis of OA in primary care

Participants with a recorded consultation for joint-related problems will be classified into those receiving an OA diagnosis recorded by their GP and those receiving a non-specific symptom code (e.g. arthralgia). The proportion of participants who (a) consult and (b) are diagnosed with OA will be described using simple descriptive statistics. Logistic regression will be used to identify which features, including OA phenotype, are strongly associated with consultation and foot OA-diagnosis. The effect of missing primary outcome data will be investigated using multiple imputation methods.

#### Describing self-care and primary care

Annual consultation rates and cumulative consultation probabilities will be calculated over the three-year period. Using logistic regression and survival analysis techniques, we will investigate further how different phenotypes relate to subsequent patterns of primary care consultation (for joint pain, other morbidities, and specifically for OA) and referral to secondary care. Self-care reported by participants in the surveys at each 18 month time-point will be described.

#### Modelling the outcomes of care

We will model the effects of care on impairment, activity limitation and participation restriction. We aim to use propensity scores (ie the propensity or likelihood of a person to seek healthcare given their characteristics) and random effects repeated measures multilevel models in order to take into account the effects of both observed and unobserved covariates on outcome at each follow-up time point. Using consultations and secondary care referrals from medical record review, together with socio-demographic, clinical, general health and phenotype characteristics, we will apply these methods to a series of analyses in which the separate effects of each of the components of consultation on subsequent outcomes at each follow-up time point will be modelled.

## Discussion

Symptomatic foot OA is a common problem, yet is under-researched relative to other sites commonly affected by OA, such as the knee and hand [6]. In this three-year prospective epidemiological study, we will combine survey data, clinical, radiographic, and ultrasound assessment, and primary care consultation records to describe the frequency and co-occurrence of OA at frequently affected joints of the foot, and to relate their occurrence to symptoms, function, clinical examination findings and life-style factors such as footwear. We will also describe the natural history of clinical symptoms relating to foot OA and assess how these present to primary care and are subsequently diagnosed and treated.

This study will focus on OA of the foot yet, in reality, people with OA are commonly affected at multiple joint sites [23,74]: a quarter of patients awaiting knee and hip replacement surgery have generalised radiographic OA [75]. Pain and functional impairment have been shown to be greater as the number of painful joint sites increases [11,16,76]. This study has been specifically designed to complement previous clinical assessment studies of the knee [30] and hand [31], which will permit data to be combined across all three cohorts, allowing more detailed investigation of patterns of multiple joint involvement, and the comparative and additive effects of pain and OA on symptoms and outcome.

An obvious limitation of our study is that asymptomatic people will not be invited for to attend for clinical assessment, so we will not be able to estimate the frequency of asymptomatic radiographic OA or clinical examination findings. However, symptoms are the presenting feature to primary care and, as with our previous clinical assessment studies [30,31], are the starting point in this study. This enables us to investigate the occurrence of foot osteoarthritis and inter-relationship between clinical signs, symptoms, and radiographic disease within symptomatic individuals and their clinical course over time.

In phase two of this study, every effort will be made to maintain the quality of the data obtained and minimise information bias in the data that will be collected at the research clinics. Standardised interview questions and physical assessment protocols have been developed and are described in detail in an Assessor Manual which will be given to each Research Assessor. Research Assessors will undergo a period of training prior to the start of the study. Quality control will be reviewed at regular intervals throughout the course of the study to ensure continued adherence to the protocols.

## Competing interests

HBM is Editor-in-Chief of *Journal of Foot and Ankle Research*. It is journal policy that editors are removed from the peer review and editorial decision making processes for papers they have co-authored. All other authors declare that they have no competing interests.

## Authors' contributions

All authors participated in the conception and design of the study, and drafting of the manuscript. All authors read and approved the final manuscript.

## Supplementary Material

Additional file 1 **Footwear questionnaire**. Click here for file
