# Supplementary material for: The clinical assessment study of the foot (CASF): study protocol for a prospective observational study of foot pain and foot osteoarthritis in the general population
Source: J Foot Ankle Res. 2011 Sep 5;4:22. doi: 10.1186/1757-1146-4-22 (PMC3180294; doi:10.1186/1757-1146-4-22)
Supplement: Additional file 1 — Footwear questionnaire. [file 1757-1146-4-22-S1.DOCX]

This section is about the types of shoes that you wore at different stages of your life.

We are interested in two parts of your shoes: the front of the shoe and the height of the heels.

**1. The front of the shoe**

Please look at the four pictures below.

For **each period of your life**, please put a cross in the box to show which picture looks most like the shoe you wore **most of the time**.

For any age that you have not yet reached, please leave the line blank. For example if you are aged 60 to 69 years complete lines 20 to 29 years, 30 to 39 years, 40 to 49 years, 50 to 59 years and 60 to 69 years. Do not complete the lines for ages 70 to 79 years and 80 years and over.

Please think only about the **front** of the shoe.

***(Please put a cross in one box on each line)***

| Your age in years |  |  |  |  |
| --- | --- | --- | --- | --- |
| 20 to 29 | □ | □ | □ | □ |
| 30 to 39 | □ | □ | □ | □ |
| 40 to 49 | □ | □ | □ | □ |
| 50 to 59 | □ | □ | □ | □ |
| 60 to 69 | □ | □ | □ | □ |
| 70 to 79 | □ | □ | □ | □ |
| 80 and over | □ | □ | □ | □ |

**2. The height of the heel (women only)**

Please look at the four pictures below.

For **each period of your life**, please put a cross in the box to show which picture looks most like the height of the heel you wore **most of the time**.

For any age that you have not yet reached, please leave the line blank. For example if you are aged 60 to 69 years complete lines 20 to 29 years, 30 to 39 years, 40 to 49 years, 50 to 59 years and 60 to 69 years. Do not complete the lines for ages 70 to 79 years and 80 years and over.

Please think only about the **height** of the **heel, not** the shape or width of the heel.

***(Please put a cross in one box on each line)***

| Your age in years |  |  |  |  |
| --- | --- | --- | --- | --- |
| 20 to 29 | □ | □ | □ | □ |
| 30 to 39 | □ | □ | □ | □ |
| 40 to 49 | □ | □ | □ | □ |
| 50 to 59 | □ | □ | □ | □ |
| 60 to 69 | □ | □ | □ | □ |
| 70 to 79 | □ | □ | □ | □ |
| 80 and over | □ | □ | □ | □ |
